# Innovative method with two-stage surgery for Ewing sarcoma with personalized distal clavicle reconstruction: A case report and diagnosis review

**DOI:** 10.3389/fonc.2023.1134324

**Published:** 2023-03-29

**Authors:** Patcharapol Arunwatthanangkul, Panichanok Piyabenjarad, Siraprapa Wisitthipakdeekul, Thanapon Chobpenthai

**Affiliations:** Princess Srisavangavadhana College of Medicine, Chulabhorn Royal Academy, Bangkok, Thailand

**Keywords:** Ewing sarcoma, distal clavicle, personalized 3D reconstruction, patient-specific implants, clavicle reconstruction

## Abstract

A 13-year-old boy presented with a growing lump on his left clavicle for 5 months. The plain radiograph revealed an osteolytic mass with aggressive periosteal reaction, suggesting a malignant lesion. The results of advanced imaging and histopathological examinations revealed that the patient had Ewing sarcoma without metastasis. The two-stage surgery was as follows: resection–observation–reconstruction. The underlying rationale was that Ewing sarcoma has a high recurrence. After 2 years of resection, the patient had remission, and he currently has a personal 3D-printed titanium implant with intact shoulder function.

## Introduction

Ewing sarcoma is the second most common bone tumor in children and young adults (1). This type of cancer can develop in any bone but usually occurs in the lower extremities, most commonly in the pelvis and femur (2). Although occurrence in the clavicle has been reported, it is considered a rare case (3). Previous studies have reported alternative surgical techniques; however, to our knowledge, a suitable management strategy has yet to be established for Ewing sarcoma of the clavicle (4).

## Background

**Figure 1:**
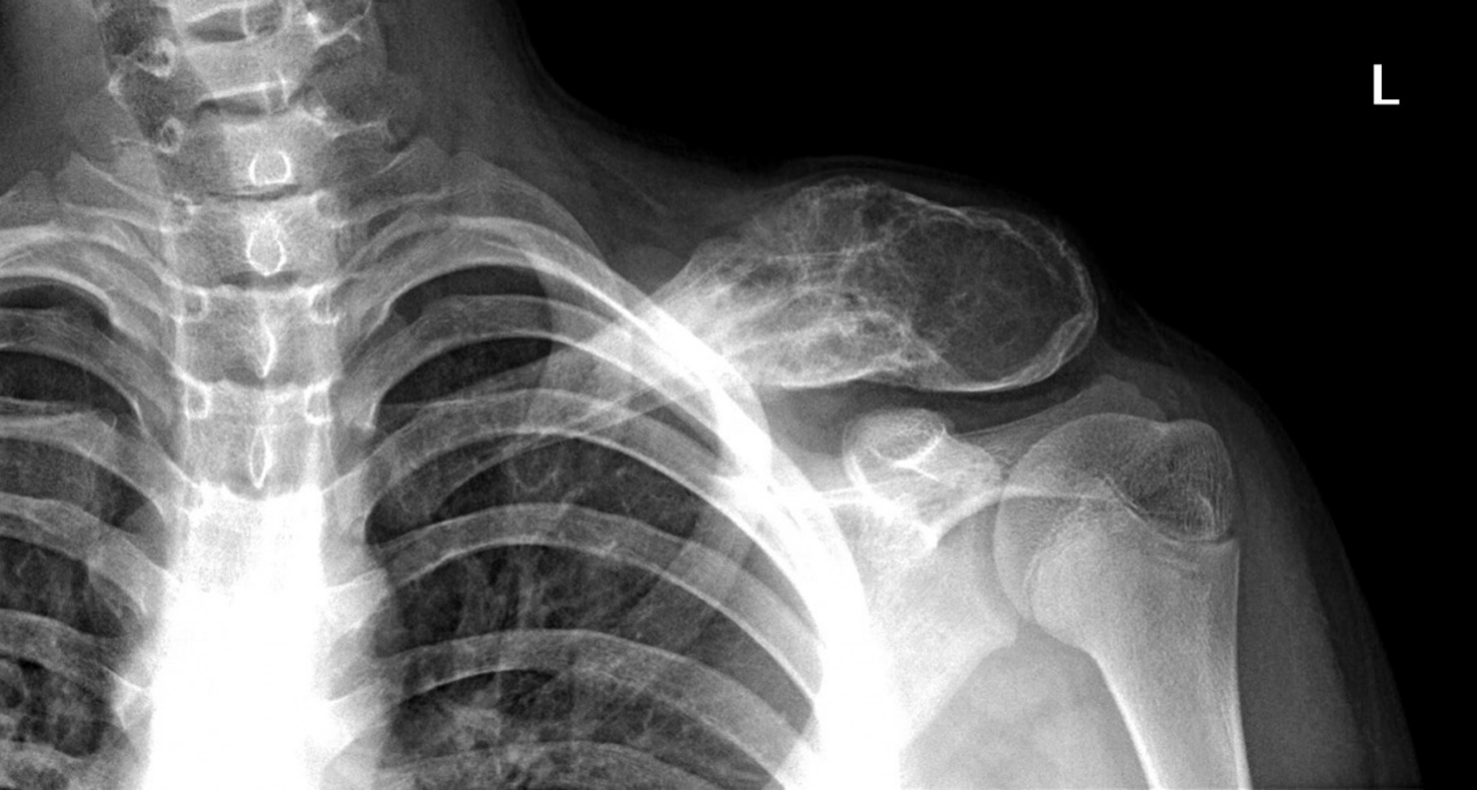
Plain radiograph of the left clavicle showing an abnormal, enlarged osteolytic lesion with a moth-eaten, lamellated appearance, and focally disrupted aggressive periosteal reaction throughout the distal half of the clavicle, with a swollen overlying soft tissue.

**Figure 2:**
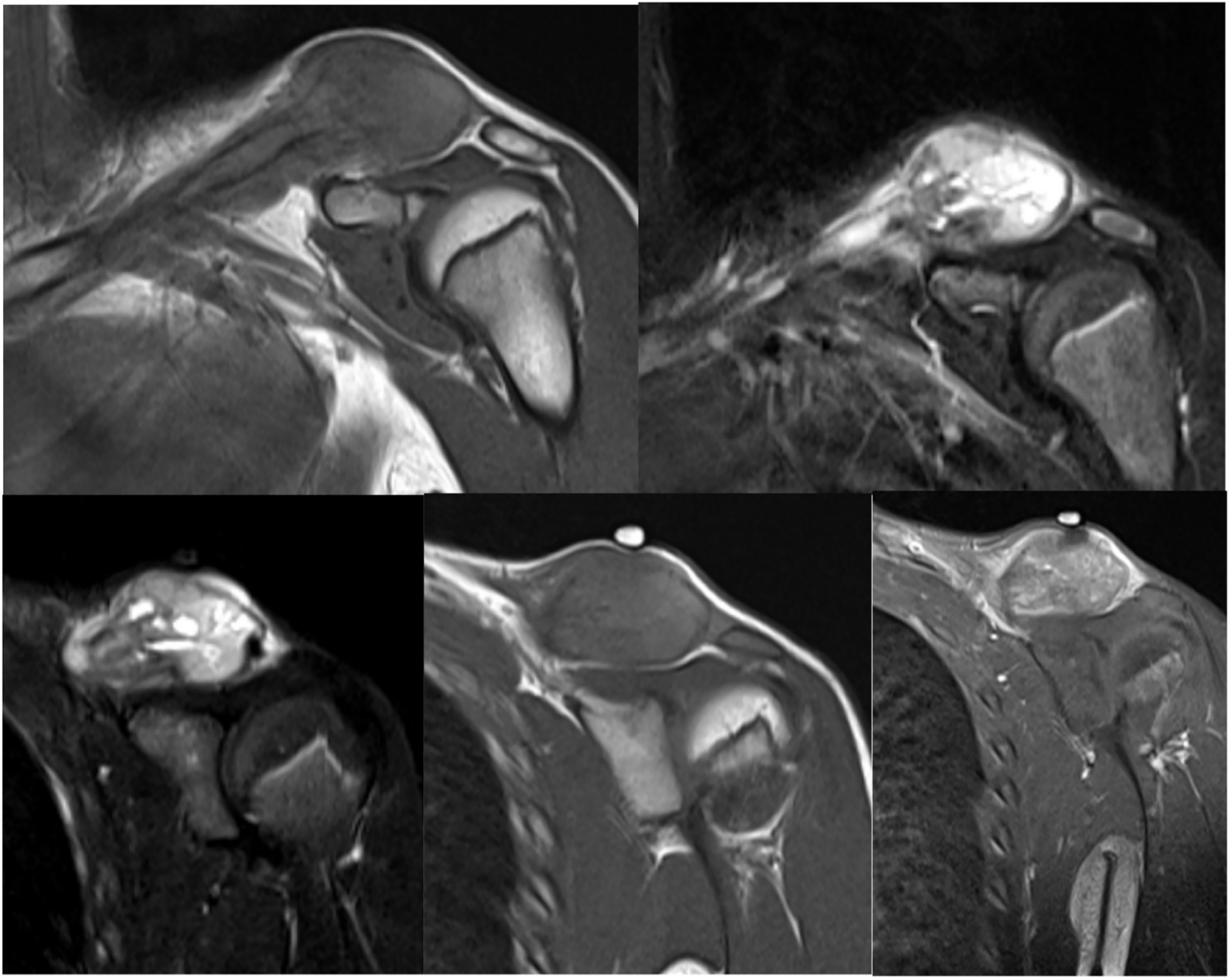
MRI of the shoulder revealing a 10.1 × 4.1 × 3.3-cm expansible intramedullary lesion on the left clavicle bone with intermediate T1 heterogeneous signal and low hyper T2 with heterogeneous enhancement, which likely corresponded to Ewing sarcoma.

A 13-year-old boy presented with a growing lump on his left clavicle for 5 months. The patient had a 10-cm painless, smooth, and palpable mass with a rubbery-to-hard consistency on his left distal clavicle. The patient’s blood reports were normal. Plain radiography of the left clavicle showed an abnormal, enlarged osteolytic lesion with a moth-eaten, lamellated appearance. It also showed a focally disrupted aggressive periosteal reaction throughout the distal half of the clavicle, with a swollen overlying soft tissue (**Figure 1**). Skeletal scintigraphy revealed increased focal tracer uptake at the left clavicle, corresponding to the primary bone lesion. The magnetic resonance imaging result showed a 10.1 × 4.1 × 3.3-cm, expansible, intramedullary lesion at the left clavicle bone with intermediate T1 heterogeneous signal and hyperintensity T2 with heterogeneous enhancement, which suggested Ewing sarcoma (**Figure 2**). No lung and whole-abdomen abnormalities were identified. The results of a histopathologic analysis showed a round cell tumor corresponding to Ewing sarcoma of the left distal clavicle.

The patient was prescribed with neoadjuvant therapy with 14 cycles of vincristine, doxorubicin, cyclophosphamide, ifosfamide, and etoposide. The first stage of operation is wide resection at the left distal clavicle and reconstruction with plate and cementation. With no evidence of recurrence noted in 2 years, the patient was subjected to the second-stage operation with a patient-specific 3D-printed personalized left distal clavicle reconstruction.

**Figure 3:**
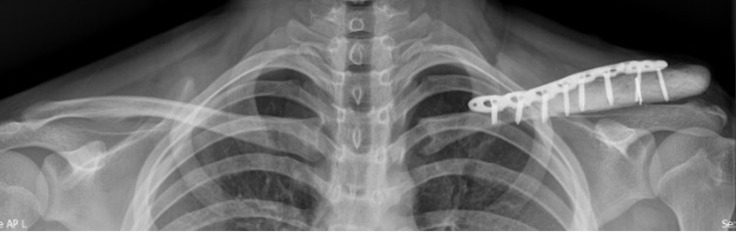
Radiograph of the left clavicle at 1 month after surgery and reconstruction. The patient showed no signs of recurrence or disease progression.

The first stage of this treatment involved the wide resection of the left distal clavicle and its subsequent reconstruction with a plate and cement. The results of a pathological analysis of the specimen collected from the intraoperative site showed neither viable nor tumor cells. The patient recovered well, and his left shoulder functions and range of motion remained intact. A year after the first surgery, the patient showed no signs of recurrent Ewing sarcoma (**Figure 3**).

**Figure 4:**
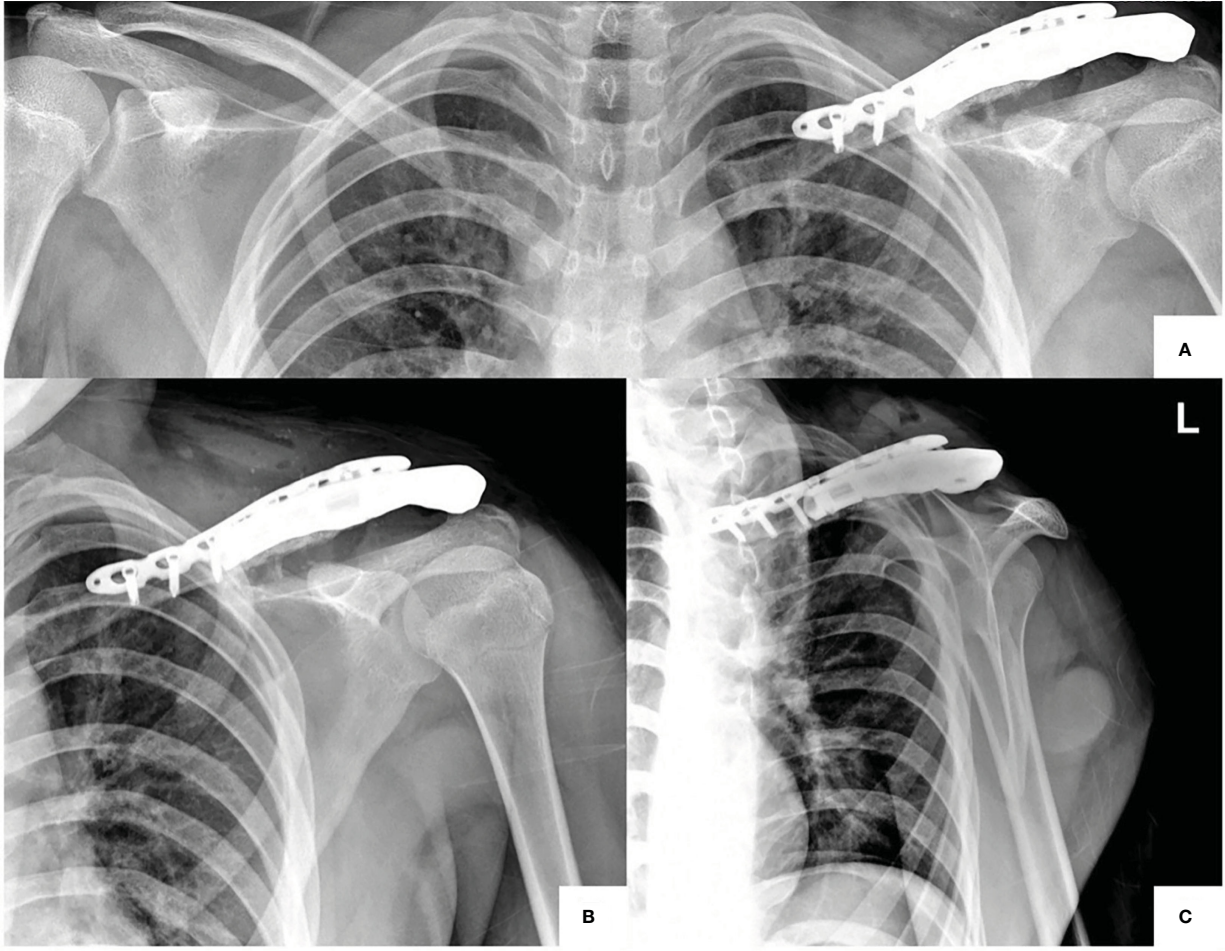
**(A–C)** Postoperative second-stage surgery with personalized 3D-printed titanium prosthesis for reconstruction. AP view, comparing both clavicles **(A)**, AP view **(B)**, and transcapular view **(C)**.

At 2 years later, the surgery’s second stage was performed with a personalized 3D-printed titanium prosthesis for reconstruction to ensure a long-term replacement. Immediate postoperative plain films were obtained again, which showed satisfactory results (**Figures 4A–C**).

At 2 months after the second-stage surgery, the wound showed good healing with minimal scarring. At 2 years after the operation, the shoulder and arm functions remained intact.

## Discussion

Osteosarcoma, the most common primary bone tumor, should be one of the first differential diagnoses for patients in this age group presenting with these symptoms. Osteomyelitis is an additional diagnosis characterized by inflammatory pain with or without an abnormal mass. However, retrospective studies and case reports have shown that Ewing sarcoma, one of the most common tumors of flat bones, is the most common tumor occurring at the clavicle (1). The children and adolescents presented with an abnormal clavicle mass. The differential diagnoses of Ewing sarcoma, osteomyelitis, and osteosarcoma were considered (**Table 1**).

**Table 1:** Differential diagnosis of children and adolescents with abnormal mass at the clavicle.

Disease	Type	Clinical	Radiographic characteristics
Ewing sarcoma	Malignant bone tumor	Local symptoms - mass, bone pain, swelling, tenderness, limping, limited function of involved area, unexplained fracture, or fracture from low-energy trauma Systemic symptoms - unintended weight loss - malaise, fatigue, fever - night sweat, night pain - lymphadenopathy - specific symptoms of metastasis organ	Plain film - osteolytic lesion, usually extended into soft tissue - poorly defined wide, permeating transition zone - aggressive periosteal reaction, lamellated onion appearance
Osteomyelitis	Inflammation and infection of bone caused by pathogenic organism	Acute Local symptoms - tenderness, erythema, swelling Systemic symptoms - fever, chill, irritability, malaise, fatigue Chronic - history of previous trauma with deep open wound - poor healing process - poor control systemic diseases such as diabetes mellitus, vascular diseases - malaise, fatigue, swelling, tenderness over the involved area	Plain film - soft tissues outlines with swelling - blurring of fat planes - regional osteopenia - vary degrees of cortical loss, bony lysis, and periosteal reaction - slow infiltrative, endosteal scalloping pattern Chronic - inhomogeneous osteosclerosis - sequestrum or cloaca (necrotic bone) formation - cortical destruction
Osteosarcoma	Malignant bone tumor	Local symptoms - mass, bone pain, swelling, tenderness, limping, limited function of involved area, unexplained fracture or fracture from low energy trauma, common site at long bone Systemic symptoms - unintended weight loss - malaise, fatigue, fever - night sweat, night pain - lymphadenopathy - specific symptoms of metastasis organ	Plain film - cortical and medullary osteolytic bone destruction - moth eaten/permeative transition zone - aggressive periosteal reaction - tumor matrix ossification and calcification, fluffy or cloud-like lesion - location usually at peri-metaphysis

The results of advanced imaging revealed the presence of a local disease at the left clavicle, likely Ewing sarcoma, without solid evidence of distant metastasis to another organ. In addition, a histopathological analysis of biopsy specimens resulted in the identification of small round cells, thus supporting the initial diagnosis that this patient had Ewing sarcoma.

A treatment protocol for Ewing sarcoma was selected following the guidelines published by Womer et al. (2012) (5). More specifically, the treatment comprised 14 cycles of alternating neoadjuvant therapy VDC/IE regimen every 2 weeks: vincristine, doxorubicin, cyclophosphamide/ifosfamide, and etoposide. The standard surgical operation for Ewing sarcoma involves a wide tumor resection with or without reconstruction. Previous studies have shown similar results and functions in total claviculectomy regardless of whether reconstruction was performed (6). The necessity of reconstruction of the clavicle after resection is still questioned, as similar functional results have been obtained in patients treated without reconstruction with a tendency to a lower incidence of complications and surgical revisions (7, 8). However, the prosthesis reconstruction was selected for esthetic reasons because it would provide the young patient with a lifelong implant instead of cementation. Previous reports on Ewing sarcoma have also attempted autograft, allograft, and cement for clavicle reconstructions in adult patients (9). Nevertheless, graft selections are limited regarding varieties and associated costs in Thailand. Owing to the high recurrence rate of this disease, it is not financially reasonable to immediately perform reconstructive surgery. Therefore, a two-stage surgery was performed: wide resection and reconstruction with medical cement, observation for possible recurrence, and definite reconstruction surgery; all procedures were explained to the patient and his family. Clavicle reconstruction with cement as a prosthesis has been previously performed as a practical and feasible short-to-medium-term therapeutic procedure with acceptable results.

A year after the first surgery, the patient showed no signs of Ewing sarcoma. His left shoulder function and range of motion remained intact and provided independence in most daily activities. However, high recurrence rates have been reported, with >70% of relapsed cases occurring approximately 24 months after diagnosis and delayed recurrence occurring 10 years after remission. In this case, remission was determined after close observations and 1-year follow-up after surgical resection. At 2 years after diagnosis and initial treatment, we then proceeded to perform the second stage of the surgery as planned.

Because of the limited graft selection mentioned above, we used a 3D-printed personalized prosthesis for reconstruction. Titanium with a hydroxyapatite surface coating was selected as the prosthetic material because it has good biocompatibility and promotes improved *in vitro* osteointegration. In the anterior chest wall reconstruction, the titanium representation is the flexible material suitable for chest wall movement (10). In addition, the implant was designed to be partially hollow to reduce the material cost and allow the insertion of a bone graft for improved osteointegration. The implant also had screw holes designed in such a way that they could fit onto an identical plate that the patient already had.

## Concluding remarks

The two-stage surgery—the recurrence observation in the first stage and then the 3D-printed personalized prosthesis for the second stage (substantial reconstruction)—provides a reasonable balance between recurrence risk and financial risk if the tumor was recurring.

## Data availability statement

The original contributions presented in the study are included in the article/Supplementary Material. Further inquiries can be directed to the corresponding author.

## Ethics statement

The studies involving human participants were reviewed and approved by Human Research Ethics Committee Chulabhorn Research Institute (project code 002/2565).

## Author contributions

All authors have read and approved the manuscript. PA and TC: Conceptualization, Methodology, Validation, Formal analysis, Investigation, Data Curation, Writing - Original Draft, Visualization, Supervision, Project administration. PP and SW: Validation, Formal analysis, Investigation, Writing - Original Draft, Supervision, Project administration, Writing - Review and Editing. All authors contributed to the article and approved the submitted version.
